# Prospection and emotional memory: how expectation affects emotional memory formation following sleep and wake

**DOI:** 10.3389/fpsyg.2014.00862

**Published:** 2014-08-04

**Authors:** Tony J. Cunningham, Alexis M. Chambers, Jessica D. Payne

**Affiliations:** Psychology, Sleep, Stress, and Memory Laboratory, Department of Psychology, University of Notre DameNotre Dame, IN, USA

**Keywords:** emotions, expectation, memory, consolidation, future relevance, sleep

## Abstract

Successful prospective memory is necessarily driven by an expectation that encoded information will be relevant in the future, leading to its preferential placement in memory storage. Like expectation, emotional salience is another type of cue that benefits human memory formation. Although separate lines of research suggest that both emotional information and information explicitly expected to be important in the future benefit memory consolidation, it is unknown how expectation affects the processing of emotional information and whether sleep, which is known to maximize memory consolidation, plays a critical role. The purpose of this study was to investigate how expectation would impact the consolidation of emotionally salient content, and whether this impact would differ across delays of sleep and wake. Participants encoded scenes containing an emotionally charged negative or neutral foreground object placed on a plausible neutral background. After encoding, half of the participants were informed they would later be tested on the scenes (expected condition), while the other half received no information about the test (unexpected condition). At recognition, following a 12-h delay of sleep or wakefulness, the scene components (objects and backgrounds) were presented separately and one at a time, and participants were asked to determine if each component was old or new. Results revealed a greater disparity for memory of negative objects over their paired neutral backgrounds for both the sleep and wake groups when the memory test was expected compared to when it was unexpected, while neutral memory remained unchanged. Analyzing each group separately, the wake group showed a threefold increase in the magnitude of this object/background trade-off for emotional scenes when the memory test was expected compared to when it was unexpected, while those who slept performed similarly across conditions. These results suggest that emotional salience and expectation cues interact to benefit emotional memory consolidation during a delay of wakefulness. The sleeping brain, however, may automatically tag emotionally salient information as important, such that explicit instruction of an upcoming memory test does not further improve memory performance.

## INTRODUCTION

Prospective memory involves the intentional formation of a memory so that information can be recalled and acted upon at a later time (Graf and Uttl, 2001). Such memories are distinguished from retrospective memories in that they are stored with the intention of being used in the future. While some prospective memory tasks require participants to perform a specific action at a specific time in the future (e.g., calling the experimenter in 1 week; Kvavilashvili and Fisher, 2007), other studies have used a future relevance cue, such as informing participants about an upcoming test, to prospectively tag information that will be useful in the future (Badets et al., 2006; Wilhelm et al., 2011). In such studies, the *expectation* that the encoded information will be needed at a later time affects memory processing, such that memory performance for the cued information is enhanced in a subsequent test compared to information thought to be irrelevant or unimportant in the future (Badets et al., 2006).

While all forms of prospective memory necessarily involve the expectation that the information will be important at a later time, other cues elicit specialized processing and storage in memory as well. For instance, emotionally salient events enjoy a privileged status in memory relative to neutral events (Kensinger and Corkin, 2003). In addition to information that is explicitly expected to be relevant in the future, emotional memories are thought to be well remembered because of their implicit future relevance to an individual’s survival (Nairne et al., 2007). In this view, while neutral information that is expected to be useful later has more obvious explicit or intentional future relevance, emotional information has “implicit” or “automatic” future relevance, meaning that this type of memory is often found to be preferentially preserved over other types of information even without explicit knowledge of future testing (Nairne et al., 2007; Payne and Kensinger, 2010; Wilhelm et al., 2011). This evolutionarily beneficial mechanism makes emotionally salient information especially potent, consuming a larger share of memory processing capacity to enhance accurate recall at a later time (see LaBar and Cabeza, 2006). Such a process may help an individual to remember and potentially avoid previously encountered dangers to increase the likelihood of survival. For instance, if we come across a deadly snake, we not only want to recognize the snake as a danger, but we also want to store a prospective memory to avoid similar snakes in the future, thus increasing our chances of survival.

There is a growing literature supporting a role for sleep in the successful consolidation of prospective memory. For example, Scullin and McDaniel (2010) had participants form a prospective memory to make a key press in response to certain target words (e.g., table or horse) that would appear in a semantic categorization task during a future session. Participants carried out this prospective task more accurately when they obtained a night of sleep in between the sessions as opposed to spending a day awake (Scullin and McDaniel, 2010). Sleep also improves memory for information that has been tagged as relevant for the future, both with emotional salience and expectation cues (Diekelmann and Born, 2010; Payne and Kensinger, 2010; Wilhelm et al., 2011). While several studies have separately investigated how expectation and emotional content are consolidated differently across delays of sleep and wake, the effect of expectation on the consolidation of emotional information remains unexplored. The purpose of the current study was to investigate how the expectation component of prospective memory affects the consolidation of emotional memories, and whether the potential relationship between expectation and emotional salience is modulated differently across periods of wake or sleep.

A period of sleep during a consolidation delay (i.e., the period of memory processing and storage that occurs following learning) better preserves memory for emotional vs. neutral information compared to an equivalent period of wakefulness, benefitting memory for negative narratives, negative images, and humorous cartoons (Wagner et al., 2001; Hu et al., 2006; Payne et al., 2008; Nishida et al., 2009; Chambers and Payne, 2014a). However, emotional memories are not always preserved in their entirety. This is reflected in studies demonstrating a role for sleep in the selective enhancement of emotional components of a memory at the expense of neutral components (hence the term “trade-off” effect, e.g., Kensinger et al., 2006; Payne et al., 2008; Payne and Kensinger, 2010; Cunningham et al., 2014). For example, Payne et al. (2008) required participants to incidentally encode scenes consisting of a negative or neutral foreground object placed on a neutral background (e.g., a snake vs. a chipmunk placed on a forest background). After a period of nocturnal sleep or daytime wakefulness, participants were tested on their memory for the objects and backgrounds, which were separated and intermixed with new images. When sleep occurred during the retention delay, the emotional memory trade-off effect was enhanced; not only was memory for negative objects better than memory for neutral objects, but the backgrounds originally paired with the negative objects were also remembered more poorly than those paired with neutral objects (Payne et al., 2008). In fact, compared to both a brief (30-min) delay and a full day of wakefulness, memory for emotional objects was the only type of memory that was benefited by sleep. All other forms of memory (neutral objects, backgrounds paired with both emotional and neutral objects) deteriorated over time. Such research speaks to the preferential manner in which sleep targets emotionally salient information for processing during the consolidation delay, especially given the fact that participants are typically not aware that future memory testing will take place (Payne et al., 2008, 2012).

These incidental emotional memory designs are different from forms of learning that benefit from sleep-dependent processing when there is explicit knowledge that new learning is occurring, or when instructions are given explicitly stating how the information will be needed in the future (e.g., successful prospection, reward scenarios, etc.). For example, overt attempts to learn a new skill, such as a procedural task, lead to increased sleep-based improvements of later performance compared to when the learning of such tasks is implicit (Robertson et al., 2004; Diekelmann and Born, 2010). In one naturalistic study, “good” sleepers (determined by measures of sleep onset latency, total sleep time, wake after sleep onset, sleep efficiency, and number of awakenings) outperformed “bad” sleepers on a prospective memory task in which participants were explicitly told to remember to hit an event-marker button on their actigraphy wrist watches before getting out of bed upon awakening (Fabbri et al., 2014). Assigning other forms of future relevance to information, such as informing participants they can receive a reward for good performance, often produces similar findings. For instance, when participants were informed following an initial learning episode that they would receive a monetary reward if they improved on a motor skill task, obtaining sleep during the consolidation delay provided a greater benefit to performance than wakefulness (Fischer and Born, 2009).

Simply expecting an upcoming memory test can be enough to improve performance as well. Across two studies, Wilhelm et al. (2011) investigated how the expectation of future testing on a word-pair associate, visuospatial (object location), and a procedural (finger tapping) task impacted performance on such tasks after a period of wake or sleep. They found that when participants were informed of the upcoming test following the learning phase, sleep benefitted later memory performance on all three tasks, while those who remained awake showed no improvement. Such effects likely rely on hippocampal processing (Gais et al., 2002; Marshall et al., 2006), which may not only support the ability to use memory to enhance future performance (Buckner, 2010; Schacter et al., 2012), but also interact with areas of the prefrontal cortex to produce specialized sleep-dependent processing of memories that are relevant to an individual’s future (Diekelmann and Born, 2010).

While both expectation and emotional salience have been shown to independently benefit memory, it is unclear whether these two memory cues interact during a consolidation delay. Sleep-dependent processing has an impact on each cue individually, improving performance over wakefulness (e.g., Payne et al., 2008; Wilhelm et al., 2011). However, it is unknown whether processing during sleep and wakefulness differentially impact memory consolidation when these two cues co-occur. It is possible that when combined, the two cues interact additively to further enhance the effects of sleep on emotional memory. Alternatively, given the purported survival value of memory benefits for emotionally salient information (Payne and Kensinger, 2010) and the possibility that memory serves an adaptive functional purpose (Nairne and Pandeirada, 2010), it is also possible that emotional salience represents such a powerful future relevance cue that the addition of explicit expectation does not further alter memory performance.

The principal goal of our study was to explore how emotional salience interacts with an expectation cue during wake-filled and sleep-filled consolidation delays. Specifically, participants encoded intact negative and neutral scenes prior to a period of sleep or wakefulness. We utilized negative scenes to represent information that was high in emotional salience as determined by subjective ratings of valence and arousal. After encoding, half of the participants in each group were told that they would be tested on their memory for the images, while the other half were not informed. This design allowed us to capitalize on the automatic enhancement of emotional memory previously shown during sleep (Payne et al., 2008, 2012; Payne and Kensinger, 2010, 2011; Bennion et al., 2013), while also manipulating the expectation of how the information will be used in the future during the consolidation period. At recognition, objects and backgrounds were presented separately and one at a time. Previous studies found an increase in memory for emotional objects at the cost of their paired neutral backgrounds and that the magnitude of this emotional trade-off effect is exacerbated over a delay of sleep (Payne et al., 2008, 2012; Payne and Kensinger, 2010, 2011).

We hypothesized that an expectation cue would change memory performance such that the disparity between memory for negatively arousing objects and memory for neutral background details (i.e., the magnitude of the trade-off effect) would be enhanced, while there would be no change in the memory pattern for the neutral scenes. We also expected that emotional salience and expectation cues would interact differently across delays of sleep and wakefulness. As in our prior studies (e.g., Payne et al., 2008), we anticipated that the sleeping brain would automatically attribute future relevance to emotional information due to its survival value, such that explicit knowledge of the test would offer no further benefit to memory following sleep. This would lead to similar magnitudes of the trade-off effect after sleep regardless of whether participants expected a later memory test. However, expectation of the test would likely increase the emotional memory trade-off following wakefulness as those who remain awake do not receive the added benefit of preferential emotional processing during sleep.

## MATERIALS AND METHODS

### PARTICIPANTS

Eighty-four University of Notre Dame students participated for payment or class credit. The University of Notre Dame Institutional Review Board approved all testing procedures, ensuring that all protocols met regulatory standards. Written consent was obtained before participation in the experiment. All participants were instructed to refrain from tobacco, caffeine, alcohol, and recreational drugs for 24 h before and throughout the study. They were fluent English speakers and had normal or corrected-to-normal vision. Participants were free of disabilities that would lead to disturbed sleep, substance abuse, major mental illness, sleep aid medications and other medications that affect the central nervous system. Four participants were excluded from analysis due to equipment failure. Thus, eighty participants (female = 41) were included in the final analyses (wake group = 41, sleep group = 39). Each group was further divided into “expectation” vs. “no expectation” groups based on instructions given to participants after the encoding task (wake group: expectation = 20, sleep group: expectation = 21).

### MATERIALS

#### Encoding materials

Participants incidentally encoded a set of 68 complex scenes that portrayed negatively arousing (e.g., a car accident) or neutral, non-arousing (e.g., a taxi cab) objects placed on plausible neutral backgrounds (e.g., a street). Thirty-four of these images were negative, while the remaining 34 were neutral. To avoid the possibility that an object/background combination was inherently more memorable, four different versions of each image were created by placing one of two similar neutral objects (e.g., two varying images of a taxi cab) and one of two similar negative objects (e.g., two varying images of a car accident) on one of two neutral backgrounds (e.g., two varying images of a street) in order to create four different but related scenes (Payne et al., 2008, 2012). These image versions were used to create four different lists of 68 scenes each, which were counterbalanced across participants. Each participant saw only one list at encoding, which was randomly determined using a mixed Latin Square design. The images within each list were also presented randomly to avoid order effects.

#### Recognition materials

At recognition, participants were presented with previously studied items (“old”) and foils that were entirely new and had never been seen before (“new”). Objects and backgrounds were presented separately and one at a time, in random order. Items in each recognition test included 34 “old” neutral objects, 34 “old” negative objects, 34 “old” backgrounds previously shown with a neutral object, 34 “old” backgrounds previously shown with a negative object, 34 “new” neutral and negative objects, and 34 “new” backgrounds (all neutral and unpaired with previously viewed objects). In total, participants saw 204 items (objects or backgrounds) during the recognition task.

Images used in the present study (studied and foil images) were previously normed for valence and arousal, using 7-point scales (*n* = 24, female = 13, mean age = 19.9, by Kensinger et al., 2006). As in our prior research (e.g., Payne et al., 2008, 2012; Payne and Kensinger, 2010, 2011; Waring et al., 2010; Steinberger et al., 2011), negative, arousing scenes were used to study the memory processing of emotionally salient information. These scenes were differentiated from neutral scenes through subjective ratings of valence and arousal. Negative images had received arousal ratings of 5–7 (with high scores representing an arousing image) and valence ratings lower than 3 (with low scores representing a negative image). All neutral items (objects and backgrounds) had been rated as non-arousing (arousal values lower than 4) and neutral (valence ratings between 3 and 5). Subjective ratings taken from our own participants at encoding verified that negative scenes were rated as low in valence (*M* = 2.7 ± 0.37) and high in arousal (*M* = 5.0 ± 0.44), while neutral scenes were rated as non-arousing (*M* = 4.4 ± 0.30) and neutral (*M* = 3.9 ± 0.40), confirming the manipulation of emotional salience. This rating task was used both to assess subjective ratings of valence and arousal and to maximize attention at encoding.

### EQUIPMENT

To perform the encoding and recognition tasks, participants were escorted to a soundproof viewing booth where they were fitted with noise-reducing headphones to minimize distraction. In the viewing booth, the images were presented using E-Prime (Psychology Software Tools) on a rear-projected 64-inch screen located 59-inch away from the participant using a NEC NP40 projector. All data analysis was completed in IBM SPSS Statistics 19.

### PROCEDURE

#### Wake condition

**FIGURE 1:**
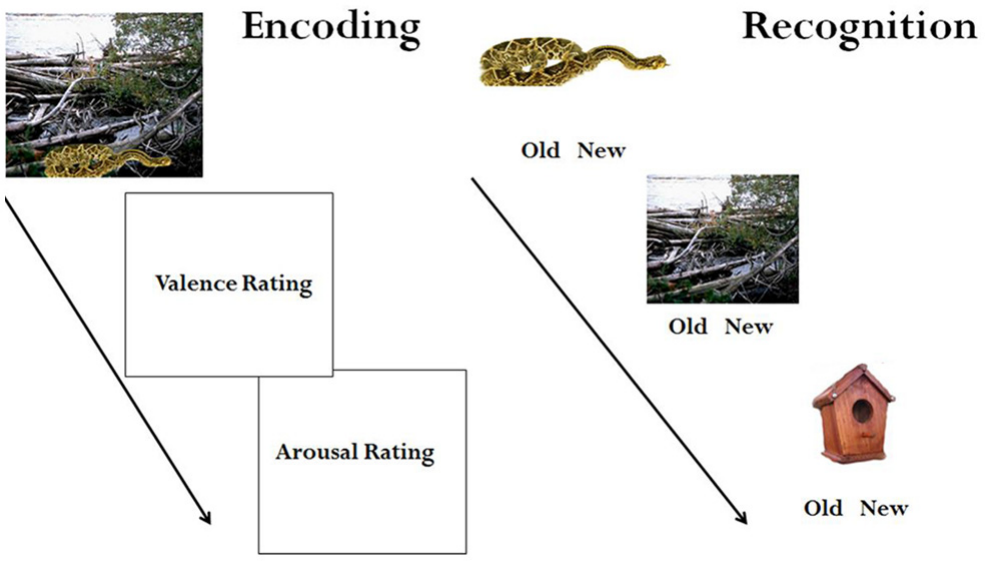
**Experimental design of Encoding and Recognition sessions**.

Participants completed the encoding session between 8:30 am and 10:00 am. Prior to starting the task, subjects were given time to acclimate to the viewing booth. Once the task began they were instructed to direct their attention to each scene for the entire duration that it was on the screen. The task began with a practice picture that allowed participants to become familiar with the rating scales and ensured that they understood the procedure. After the practice trial, each participant saw the 68 scenes presented in a random order, each displayed for 6000 ms. After the scene was removed from the display, the subject was prompted to make their ratings of valence and arousal, using scales from 1 (positive) to 7 (negative) for valence and 1 (calming or subduing) to 7 (excited or agitated) for arousal (see **Figure 1**). Upon completion of the encoding task, participants assigned to the “expected” group (*n* = 21) were given the following instructions: “When you return after an approximate 12 h retention period, we are going to have you do a memory test based on the photographs that you just viewed. The setup will be very similar to the way things went during this session, however a different display of pictures will be presented and you will be asked to determine if they are ‘old’ or ‘new’.” These instructions were given to assign explicit expectation of future relevance to the stimuli previously viewed. Those in the “unexpected” group (*n* = 20) were given no such instruction. All participants were then given packets of questionnaires to fill out for 15 min to prevent immediate rehearsal (Sorgatz and Dannel, 1978). These packets included the Stanford Sleepiness Scale (SSS; Hoddes et al., 1973), the State-Trait Anxiety Inventory (STAI; Spielberger, 2010), the Positive and Negative Affect Schedule (PANAS; Watson et al., 1988), the Beck Depression Inventory-II (BDI-II; Dozois et al., 1998), the Beck Anxiety Inventory (BAI; Beck et al., 1988) and the Mood and Anxiety Symptoms Questionnaire (MASQ; Watson and Clark, 1991). After the 15 min period all participants were dismissed from the encoding session. Wake participants were encouraged to go about their typical weekday routine but were instructed not to nap. This design allowed us to specifically target any effects of the expectation manipulation to the consolidation period between encoding and recognition (Wilhelm et al., 2011).

Participants returned to the lab between 8:30 pm and 10:00 pm on the same day (12 h after their initial session). Those that were previously informed of the test were reminded that they would be completing a recognition task, while those that had not received further instruction were informed that they would be participating in an unexpected memory test. They were escorted to the same viewing booth and again equipped with noise-reducing headphones to complete the task. Images were again presented for 6000 ms, but instead of viewing the intact images again, participants saw the objects and backgrounds presented separately, and one at a time. Participants indicated whether each item (i.e., object or background) was old or new (see **Figure 1**). After completion of the memory task, participants were debriefed, compensated, and dismissed.

#### Sleep condition

The sleep procedure was identical to the wake protocol, except participants arrived between 8:00 pm and 10:00 pm in the evening for the encoding session and completed the recognition task 12 h later between 8:00 am and 10:00 am following a night of non-invasive polysomnography-recorded sleep in the laboratory (the sleep data are the focus of another study). Once again approximately half of the sleep group was informed of the recognition test after encoding (*n* = 21), while the remaining did not expect the memory test (*n* = 18).

### DATA ANALYSIS

Subjective ratings of the scenes were assessed as the mean rating of valence and arousal during the encoding session, and these scores were calculated both across all participants and separately for each condition (sleep vs. wake; see **Table 1**).

**Table 1:** Average subjective and visceral reactivity to negative and neutral scenes at encoding.

*Subjective ratings encoding*						
	Stimuli type			
	Negative		Neutral		Difference	
Measurement type	Mean	SD	Mean	SD	*t*	*p*	
*All participants*
Valence ratings	2.71	0.37	4.43	0.30	32.70	<0.001*
Arousal ratings	5.00	0.44	3.85	0.40	18.58	<0.001*

	**Group**			
	**Sleep (*n* = 39)**		**Wake (*n* = 41)**		**Difference**	
**Measurement type**	**Mean**	**SD**	**Mean**	**SD**	***t***	***p***

*Negative stimuli*
Valence ratings	2.75	0.36	2.67	0.39	0.95	0.35
Arousal ratings	5.00	0.39	4.87	0.31	1.80	0.08
*Neutral stimuli*
Valence ratings	4.43	0.29	4.42	0.46	0.18	0.86
Arousal ratings	3.88	0.40	3.83	0.40	0.57	0.58

Valence was measured on a Likert scale from 1 (negative) to 7 (positive). Arousal was measured on a Likert scale from 1 (calming/relaxing) to 7 (agitating/exciting). *p < 0.001.

Overall memory retention was calculated separately for each valence (negative and neutral) and scene component (objects and backgrounds) as the number of items correctly remembered (i.e., hits) divided by the total number of items originally viewed, resulting in four separate memory score categories: negative objects, neutral objects, negative backgrounds (i.e., backgrounds originally paired with negative objects) and neutral backgrounds (i.e., backgrounds originally paired with neutral objects). As in several previous studies (Payne et al., 2012) we corrected for response bias by subtracting the proportion of false alarms (“old” judgments to new pictures) from the overall memory retention score to calculate final memory scores for each valence and scene component. For our purposes, “memory trade-off” was defined as the difference between object and background memory. To calculate the memory trade-off score we created difference scores by subtracting the corresponding background memory score from the object memory score for each valence (e.g., negative objects – negative backgrounds) within each group (sleep and wake). For example, if a person’s corrected negative object score was 0.70 and their corrected negative background score was 0.50, their memory trade-off score would be 0.20.

## RESULTS

### SUBJECTIVE RATINGS AT BASELINE

Using baseline measures generated during initial encoding of the images, we first confirmed that the negative scenes were perceived as being more emotionally salient than the neutral scenes. Across all participants, paired sample *t*-tests revealed that negative scenes were subjectively rated as being more negative [*t*(79) = 32.7, *p* < 0.001] and more arousing [*t*(79) = 18.6, *p* < 0.001] than neutral scenes. Independent sample *t*-tests confirmed that the wake and sleep groups did not differ in their subjective ratings of valence and arousal for negative or neutral scenes (see **Table 1**).

### MEMORY PERFORMANCE

We first conducted a 2 (valence: negative, neutral) × 2 (scene component: object, background) × 2 (instruction: expected, unexpected) × 2 (group: sleep, wake) mixed ANOVA, with valence and scene component as the repeated measures. This analysis revealed a significant interaction between instruction and scene component [*F*_1,76_ = 4.7, *p* = 0.03], which was driven by a greater difference between scene components (objects vs. backgrounds) when the memory test was expected compared to when there was no expectation of future testing. This finding demonstrates that expectation alone had an effect on memory regardless of valence. There was also a significant interaction between valence and scene component [*F*_1,76_ = 70.9, *p* < 0.001], driven by a significantly greater difference between scene components (objects vs. backgrounds) for emotionally salient, negative scenes compared to the non-arousing, neutral scenes. This result provides additional support for the emotional memory trade-off effect (e.g., Kensinger et al., 2007; Payne et al., 2008).

**FIGURE 2:**
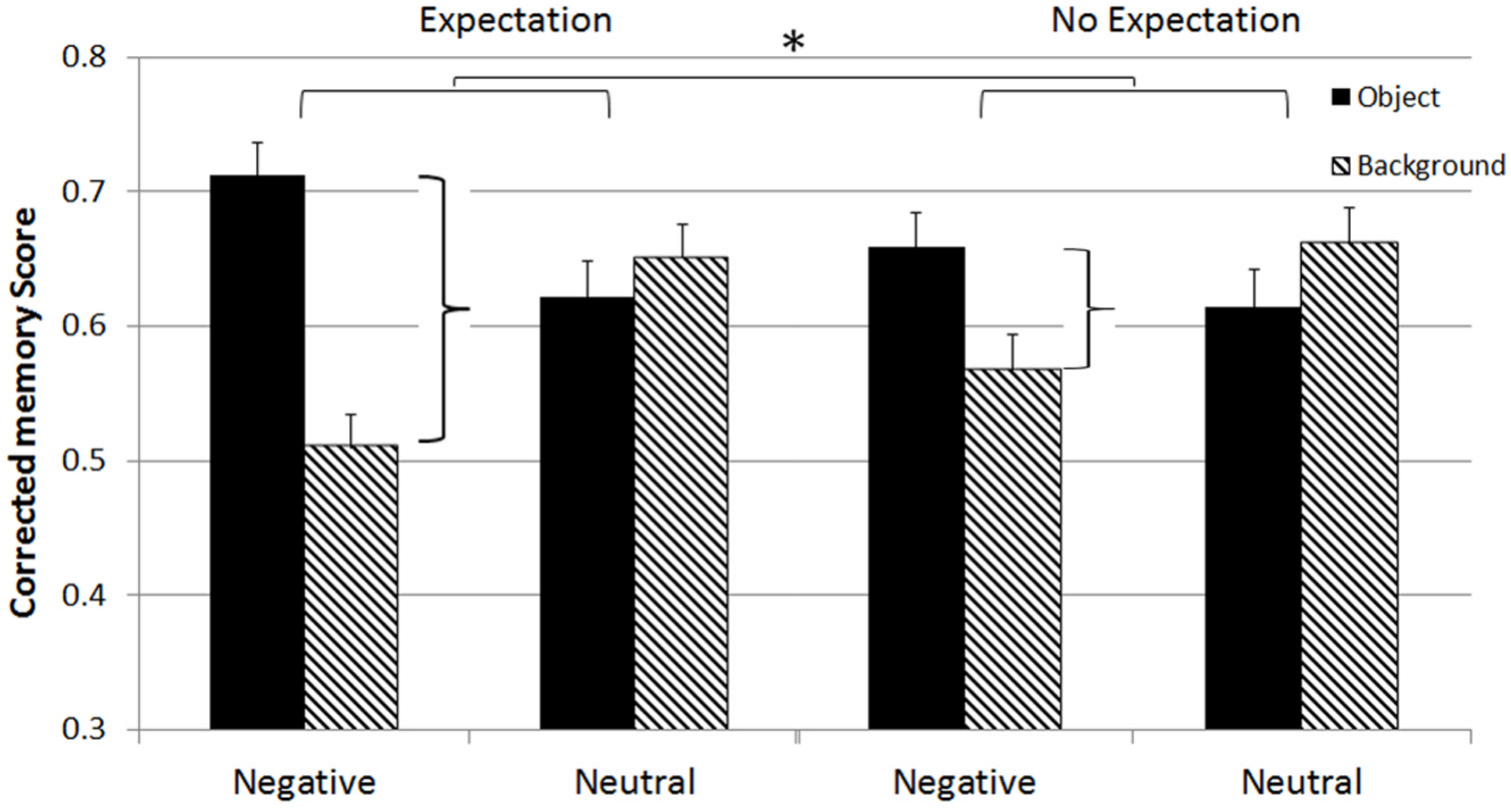
**Expectation × Valence × Scene component three-way interaction.** *p < 0.05.

Importantly, there was also a significant three-way interaction among valence, scene component, and instruction [*F*_1,76_ = 4.3, *p* = 0.04; see **Figure 2**] supporting our hypothesis that the expectation manipulation influenced the emotional memory trade-off effect. To probe this interaction further we created trade-off magnitude scores by calculating the difference between memory for objects and memory for their associated backgrounds, for both negative and neutral scenes (as described in Data Analysis). A 2 (valence: negative, neutral) × 2 (instruction: expected, unexpected) mixed ANOVA revealed a main effect of expectation [*F*_1,78_ = 5.0, *p* = 0.03]. There was also a significant two-way interaction of valence and instruction [*F*_1,78_ = 4.3, *p* = 0.04]. Independent sample *t*-tests on these scores on the combined sleep and wake groups revealed that this interaction was driven by more than a twofold increase in memory trade-off magnitude for negative scenes (objects vs. backgrounds) when participants expected the memory test compared to when it was unexpected [*t*(78) = 2.6, *p* = 0.01; see **Figure 2**, first and third clustered columns]. Concurrently, memory trade-off scores for neutral information remain the same across expectation conditions [*t*(78) = 0.76, *p* = 0.45].

**FIGURE 3:**
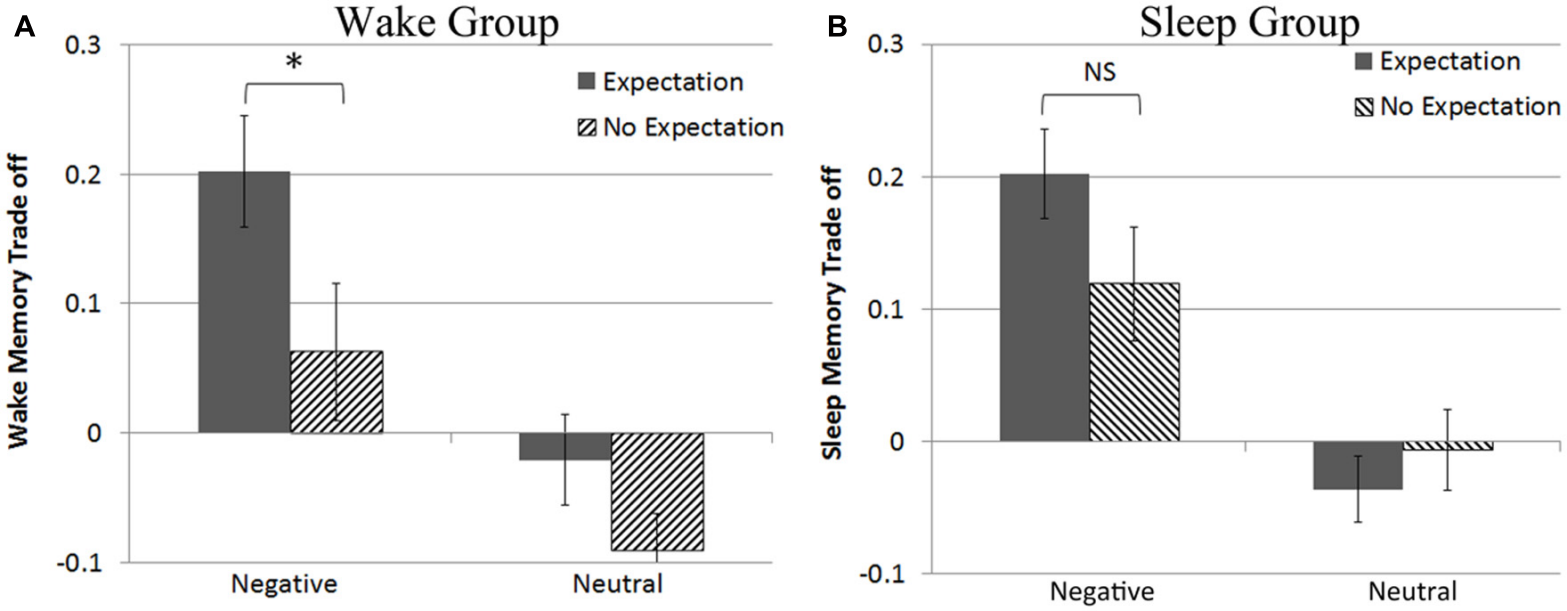
**(A)** Expectation increases negative memory trade-off in the wake group **(B)** but has no effect after a delay of sleep. *p < 0.05.

To explore our *a priori* hypothesis that emotional salience and expectation cues would differently affect memory in the sleep and wake groups, we examined the memory trade-off magnitude scores for negative and neutral scenes separately in the sleep and wake groups as a function of expectation. When the subsequent test was *unexpected* we found a significant increase in memory for negative objects compared to the backgrounds on which they were placed for those who slept [*t*(17) = 2.8, *p* = 0.01] but not for those who remained awake [*t*(20) = 1.2, *p* = 0.25], replicating previous findings (Payne et al., 2008). Interestingly, when participants *expected* the recognition test at the second session, this emotional memory trade-off was seen in both the sleep [*t*(20) = 6.0, *p* < 0.001] and the wake groups [*t*(19) = 4.7, *p* < 0.001]. In fact, in the wake group, there was a significant threefold increase in the size of the trade-off for negative scenes when the memory test was expected compared to when the participants did not expect the future testing [*t*(39) = 2.02, *p* = 0.05; see **Figure 3A**]. When participants were allowed to sleep during the consolidation delay, trade-off magnitude scores remained equivalent for negative scene components regardless of expectation condition [*t*(37) = 1.5, *p* = 0.14; see **Figure 3B**]. These results support our secondary hypothesis that that expectation cues do interact with emotional salience to increase the emotional memory trade-off effect when participants remain awake, but during sleep, processes appear to identify emotional information as important for later memory, such that the expectation of a later test does not increase preferential memory for emotional objects over their paired neutral backgrounds.

## DISCUSSION

Both emotional salience and expectation are future relevance cues that have been shown to enhance memory consolidation, especially if the retention interval includes a period of sleep (Diekelmann and Born, 2010; Payne and Kensinger, 2010; Wilhelm et al., 2011). Our primary goal was to determine how expectation would affect information that is implicitly tagged with emotional salience. To do this, we examined the automatic enhancement of emotionally salient information by sleep using the emotional memory trade-off task (Payne et al., 2008) while also manipulating the explicit tagging of information as important for a later memory test following encoding. We found that the addition of expectation to the consolidation of negative and neutral scenes enhanced the preferential storage of emotional objects over their paired neutral backgrounds, while having no effect on memory for the neutral scenes. Based on prior research (Payne et al., 2008; Wilhelm et al., 2011), we also expected emotional salience and expectation cues to interact differently across consolidation delays of sleep and wake. Participants who remained awake during the consolidation period showed a dramatic increase in the magnitude of the emotional memory trade-off effect when they were informed of the test compared to when they were not informed, a finding which further corroborates the beneficial influence of expectation on memory (Badets et al., 2006). Critically, those who slept during the delay performed similarly regardless of whether they received explicit information about the later memory test or if the subsequent test was a surprise. This suggests that sleep alone does a sufficient job of tagging emotionally salient information as important for later memory, such that expectation of an upcoming memory test for that information does not further enhance performance. However, when participants do not receive the benefit of sleep during consolidation, tagging the information with expectation cues allows for nearly equivalent emotional memory processing between the sleep and wake groups.

Although many memory studies focus on memory for the past, a growing literature has begun to examine how memory can be applied to the future (Buckner and Carroll, 2006). It has been suggested that a similar brain network consisting of frontal and medial temporal lobe structures supports the ability to use memory retrospectively and prospectively (Buckner and Carroll, 2006). Consistent with this notion, when asked to imagine episodic details of a possible future event in which they might engage, participants consistently recruit brain regions also important for remembering the past (Addis and Schacter, 2012). This includes the hippocampus, which may be necessary for the recombination of segments of memories to build future simulations and thus predict future events (Schacter et al., 2007; Buckner, 2010). Given such findings, it makes sense that sleep, which has known influences on retrospective memory tasks relying on the hippocampus (Diekelmann and Born, 2010), also influences memory for information needed in the future, including prospective actions (Scullin and McDaniel, 2010), emotionally salient material that may be relevant for survival (Payne et al., 2008), and information expected to be important (Wilhelm et al., 2011).

While previous evidence suggests that both emotional salience (Hu et al., 2006; Payne et al., 2008; Nishida et al., 2009) and expectation cues (Wilhelm et al., 2011) individually bolster performance following sleep, this is the first study to manipulate both of these factors simultaneously. Given that we found the test instruction manipulation to impact sleep and wake groups differently, it is possible that the effect of future relevance type (i.e., emotional salience vs. expectation) on later memory ability depends on the state of the brain during the consolidation interval. Consolidation processes active during sleep may preferentially rely on the emotional salience of the learned information to tag information as important, with no added benefit of expectation tags. Processes occurring during wakefulness, on the other hand, appear to require that the information be given *explicit* instruction in order for the same level of memory processing to occur. This explicit instruction may induce increased rehearsal of the emotional content of scenes during the waking delay, thus benefiting memory for the negative object over neutral information in the background. This rehearsal may be equivalent to the processing that naturally occurs during sleep. The unique neurobiology of the sleeping brain (specifically during REM sleep) is thought to be optimally suited for reprocessing emotional information due to the heightened activation of brain regions implicated in emotional memory consolidation (including the amygdala; Braun et al., 1997), as well as the increase in neuromodulators known to support emotional memory formation (acetylcholine, cortisol; Plihal and Born, 1999; Stickgold, 2005; Payne and Kensinger, 2010; Payne, 2011). These features may make REM sleep the optimal brain state for processing emotionally relevant information over other types of relevant information. Such processing may take priority during sleep, which is adaptive given the purported survival value of emotional information (Payne and Kensinger, 2010), and may overshadow the processing of other types of memory cues. Because sleep stages associated with memory performance were not addressed in the present study, it is unknown if these effects may be related to REM sleep obtained over the course of the night. However, given the importance placed on this stage in previous study of sleep-dependent emotional memory consolidation (Nishida et al., 2009; Payne et al., 2012), work to investigate this topic is already underway.

The results obtained in the current study following the wake delay stand in contrast to some previous work, however, which found no difference in memory performance between those who expected or did not expect a later test and remained awake during the consolidation delay (Wilhelm et al., 2011). In this study, knowledge of the upcoming test was similarly manipulated, with half of the participants informed of an upcoming test following learning, and prior to a sleep or wake delay. However, different from our methods, Wilhelm et al. (2011) utilized cued recall rather than recognition to test memory for non-emotional word-pairs learned at encoding. The participants were also trained to a 60% correct response criterion at initial learning, and word-pairs were semantically related. It is possible that these differences in the type of information learned and the means of testing memory underlie the different findings between studies. Such features of the learning event could have provided other mnemonic cues to rely on (e.g., semantic relationships), thus potentially making the memory test easier and overshadowing the expectation processing that occurs during wakefulness. Future work will be necessary to examine the exact nature of this type of processing and under what circumstances it operates.

One caveat is that while both sleep groups performed statistically similarly in the emotional memory task in the present study, the group that was explicitly informed about the upcoming test showed a numerically larger increase in the emotional memory trade-off than the sleep group that was uninformed of the upcoming memory test. Similarly, the wake group showed a non-significant enhancement for negative over neutral information even when they were not informed of the upcoming memory test (similar to Payne et al., 2008), and this effect was greatly exacerbated when the information was expected to be relevant in the future. The direction of these results could indicate that emotional salience and expectation do have a slight additive or interactive effect. However, given that the current study does not statistically support this theory, future research should be aimed at further distinguishing these processes. It should also directly address circadian effects on memory performance. Although our participants showed no time of day differences in their subjective judgments of valence and arousal, it is nonetheless possible that time of day influenced memory in this study. While we find this unlikely given that our prior research has expressly addressed such concerns in similar experimental designs (e.g., Payne et al., 2008), we cannot completely rule out circadian influences on the memory data collected here. Finally, it will be interesting to explore how expectation interacts with positive emotional salience. This study utilized negative scenes because the trade-off effect is already well established with this material (Payne et al., 2008). While preliminary studies suggest that the trade-off effect for positive scenes may be less robust than for negative scenes (Waring and Kensinger, 2009, 2011; Chambers and Payne, 2014b), it will nevertheless be important to understand how expectation affects positive memory consolidation going forward.

## CONCLUSION

A necessary component of all successful prospective memory includes an expectation that the information or instruction being encoded will be relevant for future use. It is this expectation that preferentially preserves this information over other information that is less important. This study explored how expectation of future relevance would affect emotional memory consolidation across delays of sleep and wake. We found that we could alter emotional memory processing with the addition of a simple expectation cue. This indicates that the selective consolidation of emotional memories can be enhanced through instruction after encoding. Moreover, preliminary evidence suggests that the wake group showed a threefold increase in the magnitude of this object/background trade-off for emotional scenes when the memory test was expected compared to when it was unexpected, while those who slept performed similarly across conditions. These results suggest that emotional salience and expectation cues interact to benefit emotional memory consolidation during a delay of wakefulness. The sleeping brain, however, may automatically tag emotionally salient information as important, such that explicit instruction of an upcoming memory test does not further improve memory performance.

Our results have potential ecological relevance for our understanding of memory consolidation and how information might be best protected for later retrieval of emotional real-life situations. For instance, if a person witnesses a crime or an accident, explicitly instructing them that they may need to remember the details could enhance their ability to retain correct information for testimony in court. Although such ideas remain untested at this point, continued research will shed light on the role that expectation plays in the processing of information across periods of sleep and wakefulness, and the effect that it has on an individual’s subsequent memory and behavior.

## Conflict of Interest Statement

The authors declare that the research was conducted in the absence of any commercial or financial relationships that could be construed as a potential conflict of interest.
